# Intrinsic processes drive variability in basal melting of the Totten Glacier Ice Shelf

**DOI:** 10.1038/s41467-018-05618-2

**Published:** 2018-08-07

**Authors:** David E. Gwyther, Terence J. O’Kane, Benjamin K. Galton-Fenzi, Didier P. Monselesan, Jamin S. Greenbaum

**Affiliations:** 1 Institute for Marine and Antarctic Studies, University of Tasmania, Private Bag 129, Hobart, TAS 7001 Australia; 2 CSIRO Oceans and Atmosphere, Hobart, TAS 7001 Australia; 3 Australian Antarctic Division, Kingston, TAS 7050 Australia; 4 Antarctic Climate & Ecosystems Cooperative Research Centre, University of Tasmania, Hobart, TAS 7001 Australia; 5 Institute for Geophysics, University of Texas at Austin, Austin, TX 78758 USA

## Abstract

Over the period 2003–2008, the Totten Ice Shelf (TIS) was shown to be rapidly thinning, likely due to basal melting. However, a recent study using a longer time series found high interannual variability present in TIS surface elevation without any apparent trend. Here we show that low-frequency intrinsic ocean variability potentially accounts for a large fraction of the variability in the basal melting of TIS. Specifically, numerical ocean model simulations show that up to 44% of the modelled variability in basal melting in the 1–5 year timescale (and up to 21% in the 5–10 year timescale) is intrinsic, with a similar response to the full climate forcing. We identify the important role of intrinsic ocean variability in setting the observed interannual variation in TIS surface thickness and velocity. Our results further demonstrate the need to account for intrinsic ocean processes in the detection and attribution of change.

## Abstract (web summary)

Low frequency intrinsic ocean variability has an unknown impact on Antarctic ice shelves, yet can arise even in the absence of varying climate forcing. Here, the authors show that this variability significantly affects modelled basal melting under the Totten Ice Shelf, with implications for the attribution of change.

## Introduction

Understanding the rate of change of ice shelf thinning and the processes governing change is critical for understanding mass balance and hence the Antarctic ice sheet contribution to sea level rise. Over the period 2003–2008 (~5 years), the Totten Glacier Ice Shelf (TIS) was observed^1^ to be lowering at ~0.41 m yr^−1^. However, extending the observational satellite record to encompass the period 1994–2012 (~18 years)^2^ showed that the thickness of the TIS was subject to large periodic 3–4 year fluctuations of approximately ±40 m with almost no net thickness change (within the error bars) over the 18 year period. Further, interannual variation has been detected in surface velocity^3–5^ and the grounding line location is showing change^6^. It is not clear how much of the observed temporal change in TIS is as a result of internal glacier-ice shelf dynamics or is in response to variability in ocean-driven basal melting. We have chosen to characterise the part of melt rate variability that arises in response to variability intrinsic to the ocean. This study serves as a necessary basis for understanding and quantifying what fraction of the observed variations in melt rate are independent of natural fluctuations in the climate modes and trends arising from anthropogenic forcing.

Basal melting thermodynamically couples the ocean to the ice shelf system. As a result, ice shelf thickness responds to variability in the nearby ocean system. This variability could be externally forced, for example, by climate change, interannual and multidecadal climate modes, the annual cycle, or tides. In addition, variability could also be generated by the non-linear intrinsic ocean response to forcing, as opposed to the deterministic response from interannual atmospheric forcing. Low-frequency intrinsic ocean variability results from non-linear interactions within the ocean and in response to stochastic-to-seasonal timescale changes in atmospheric forcing^7–15^, in line with the paradigm of Stochastic Climate Models^16,17^. As a result, the ice sheet will exhibit a combined response to both intrinsic and externally forced climate variability.

Observations of basal melting and ice shelf change generally consist of short records which limit the ability to distinguish intrinsic from forced variability. Advances have been made in identifying the ice shelf response to observed low-frequency internal variations in the Southern Hemisphere climate. For example, El Niño-Southern Oscillation (ENSO) has been suggested to drive stronger cross-shelf transport of oceanic heat leading to a lowering of Amundsen Sea ice shelves^18^. Conversely, atmospheric hindcast models for 1979–2015 have suggested that ENSO has a statistically non-significant impact on the westerly winds which drive stronger cross-shelf transport^19^. This highlights the complex impact that low-frequency climate variations have on ice shelf mass loss. It also emphasises the importance of ensuring that correlations and trends are statistically significant, particularly given the short satellite record relative to the timescale of climate variations like ENSO.

Ocean models can be used to simulate and quantify variability beneath ice shelves. Idealised coupled ice sheet-ocean models subject to periodic variability in oceanic heat content have indicated the ice sheet response is dependent on the forcing periodicity in relation to the ice shelf residence time^20^. However, the origin of this periodic forcing and the response of a realistic ice shelf is uncertain.

**Fig. 1:**
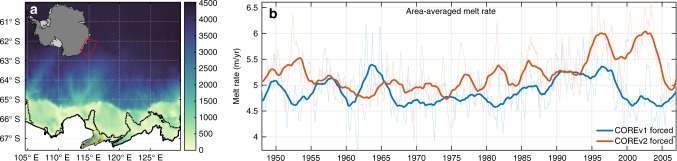
Modelled TIS basal melting. **a** Domain and model bathymetry with TIS outlined orange. **b** Area average melt rate for COREv1 repeated normal-year forcing and COREv2 interannual forcing for 1949–2007. Raw data (light coloured lines) is smoothed with a 1.5-yr filter

In this work, we present simulations of the response of basal melting beneath TIS to intrinsic ocean variability (model domain shown in Fig. 1a). We use lateral boundary conditions that contain only intrinsic ocean variability and repeated normal-year atmospheric forcing (Coordinated Ocean ice Reference Experiment version 1; COREv1)^21^ to quantify the interannual intrinsic variability that impacts basal melting. Non-linear ocean processes and the response to rapid wind changes produces a significant degree of intrinsic variability in water properties on the continental shelf and thus basal melting beneath TIS. Further simulations compare the basal melt response from only intrinsic variability to the full climate response from 1949 to 2007 (Coordinated Ocean ice Reference Experiment version 2; COREv2)^22^ and show that intrinsic ocean variability can produce a similar magnitude response to the interannual climate forcing. These results demonstrate the need to account for the contribution of intrinsic ocean processes in order to correctly detect and attribute ice shelf change.

## Results

### Melt rate interannual variability

As the COREv1 run is not forced with any interannual climate modes, any resulting low-frequency variability must be internally generated by the model or enters as intrinsic variability through the ocean boundaries. In contrast, the COREv2 run represents the response of the ice shelf to externally forced interannual variability. The COREv1 and COREv2 simulations produce a mean basal melting of 5.0 ± 0.5 m yr^−1^ (for years 30–150) and 5.2±0.5 m yr^−1^ (for years 1949–2007), respectively (Fig. 1b). This is half the glaciological inferred value^23^, however the mean spatial distributions (see Results section ‘Intrinsic variability in melt rate’) are similar to previous studies^4,24,25^. The mean lower melt rate results mainly from the cooler CORE ocean boundary conditions that introduce a constant cold bias as compared to previous modelling studies which utilised different forcing conditions (see Methods).

Both the intrinsic ocean variability model (COREv1 run) and the interannual variability model (COREv2 run) exhibit basal melting variability on a range of timescales. As the COREv1 run is forced with a repeated normal-year atmosphere and the intrinsically varying ocean resulting from the application of that atmosphere forcing, it represents the intrinsic melt rate response. In contrast, the COREv2 run contains the ice shelf response to the interannually varying ocean and atmosphere over the period 1949–2007. The ratio of COREv2 (with all natural, intrinsic, internal and anthropogenic forcings) to COREv1 (intrinsic-only) melt rate anomaly variances is 1.7. This is equivalent to a signal-to-noise ratio, where the ‘signal is the combined deterministic and intrinsic response from COREv2 and the ‘noise is the intrinsic response from COREv1 (see Methods). This highlights the potential for intrinsic processes to drive significant low-frequency variability in mean melt rates.

Quantifying changes in basal melting of TIS due to anthropogenic climate change and known climate modes (e.g. ENSO), requires a knowledge of the response due to intrinsic ocean dynamics. This study hereafter focusses on the low-frequency response of basal melting to intrinsic ocean variability. Here we are only concerned with this type of variability, noting that additional questions about the thermodynamic response of the ice shelf to the longer term climate (COREv2) are the subject of follow-up studies.

### Ocean drivers of TIS variability

**Fig. 2:**
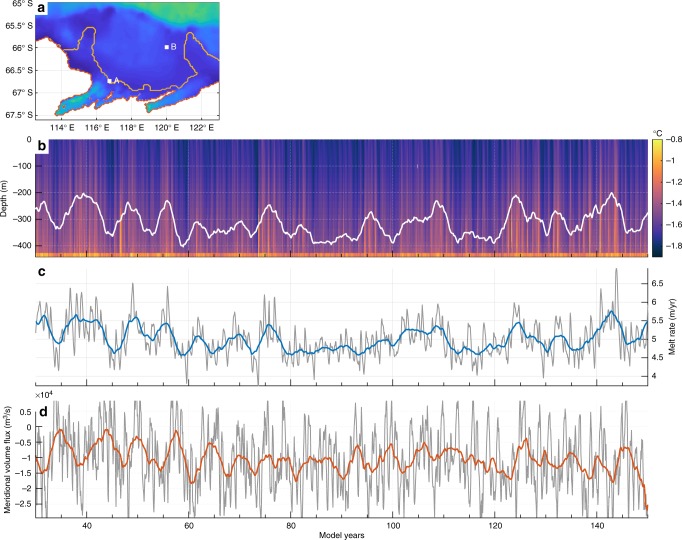
Intrinsic variability in MCDW thickness and cross-shelf exchange. **a** Intrinsic variability in ocean properties are shown at the ice front (A; 116.7° E, 66.7° S) and near the shelf break (B; 120° E, 66.0° S). **b** Potential temperature at the ice front, location marked as A, with the smoothed −1.5 °C temperature anomaly contour, representing the thickness of the bottom MCDW layer, shown in white. **c** Area-averaged melt rate for the TIS shown raw (grey line) and smoothed (blue line), and correlates well with MCDW thickness. **d** Meridional volume flux integrated over depth, with raw monthly averages (grey line) and smoothed (orange line) values. Negative values represent southward integrated transport, and hence transport onto the continental shelf

Ocean conditions at the ice shelf cavity entrance drive melt rate changes (Fig. 2a, location A). Depth-time plots of the temperature (Fig. 2b) profile show winter water (WW), the product of the seasonal sea ice formation cycle, overlaying modified circumpolar deep water (MCDW). There is also occasional formation of Antarctic surface water (not shown). These water mass properties are very similar to previous observations in this region^26–29^. The thickness of the MCDW layer, here shown by fluctuations in the −1.5 °C contour (white line in Fig. 2b), displays low-frequency variability. The interannual variability in melt rate (Fig. 2c) correlates very well with MCDW thickness (*r*-value = 0.87, with lag = 0 months) and responds linearly. This MCDW thickness variability is an intrinsic ocean response to normal-year surface forcing (wind and fluxes) and directly drives interannual variability in melt rate. Near to the continental shelf break (Fig. 2a, location B), the flow characteristics show interannual variability. This location, chosen to coincide with high variability in bottom temperature anomaly from an Empirical Orthogonal Function analysis (see Results section ‘Bottom temperature interannual variability’), shows significant interannual variability in southward depth-integrated volume flux across the grid cell, with an approximately 5–7-year period. This shows the intrinsic ocean variability associated with exchange onto the continental shelf, but the low correlation with melt rate (*r*-value = 0.02) suggests that local processes have the biggest impact on melting.

### Intrinsic variability in melt rate

**Fig. 3:**
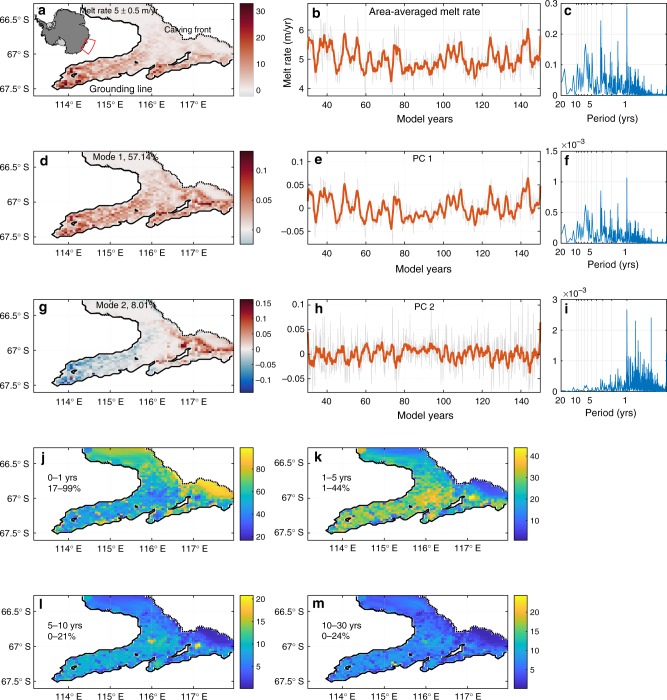
Intrinsic variability in basal melting. **a** Average melt rate (5.0 m yr^−1^ with standard deviation of 0.5 m yr^−1^) and **b** area-averaged melt rate for the Totten Ice Shelf. The first 30 years is considered model spin-up and is excluded from analysis. **c** Spectral variance (power spectrum times frequency) for the melt rate anomaly, constructed by removing the climatology. **d** Melt rate EOF mode 1 and **e** PC 1. **f** Spectral variance for melt EOF mode 1. **g** Melt EOF mode 2 and **h** PC 2. **i** Spectral variance for melt EOF mode 2. **j**–**m** SSA in-band % explained variance in melt rate, with band duration (years) and min–max variance shown. Inset in **a** shows location. Orange line in **b**, **e**, **h** is smoothed with a 1.5-yr filter, while grey line is raw data. Colour bars in **j**–**m** are scaled to the min/max variance range, as given as percentages on the left side of each panel

The area-averaged melt rate of the TIS (Fig. 3a) contains significant low-frequency variability, with a range of 4.5–6 m yr^−1^ (Fig. 3b) and power in the ~3–7 yr time band (Fig. 3c).

To identify and characterise modes of variation, we applied two different methodologies commonly used for analysing climate data (see Methods). Empirical orthogonal function (EOF) analysis identifies spatially invariant patterns in the data and accompanying time series that maximise variance. This was used to reduce the dimensionality and to decompose spatial and temporal variation into the EOF spatial modes, temporal principal components (PCs) and the variance explained by each mode. We restrict our analysis to modes explaining >5% of the variance (see Supplementary Fig. 1 for first 6 modes). The data has been linearly detrended and the climatological mean removed prior to analysis.

The EOF analysis of melt rate anomaly suggests that most variability (57% of variance) is explained by mode 1 (EOF1; Fig. 3d) which shows wide-scale coherent fluctuations in basal melting, similar to the time-mean spatial pattern in Fig. 3a. EOF mode 2 (EOF2; 8% of variance) captures enhanced shallow or deep melting, and results from water flowing either along the ice shelf front or below the ice shelf (Fig. 3g). Interannual fluctuations in average melt rate are represented strongly in the first PC of the EOF analysis (compare Fig. 3e to Fig. 3b), which contains ~3-, 5-, ~7-year and multidecadal modes (Fig. 3f). The second PC (Fig. 3h) contains mostly intra-annual variability (Fig. 3i).

While the EOF analysis captures spatial patterns of covariability it does not necessarily separate physical modes over different timescales. To achieve this, we apply singular spectrum analysis (SSA)^12,30^, which identifies spatially coherent regions of variance for a chosen time band, to analyse the leading PCs of melt rate and extract secular behaviour present at intra-annual (<1 year), interannual (1–5 and 5–10 years) and multidecadal (10–30 year) time bands (see Methods).

The in-band SSA variance shows the percentage of total variance at each location within defined time bands (Fig. 3j–m). Intra-annual modes explain ~40–50% of total variance, and closer to 90% of total variance near to the ice front (Fig. 3j). These intra-annual modes result from the seasonal cycle in sea ice production and winds. As this study focusses on interannual variability, these <1-year modes will not be further discussed. Modes with a period of 1–5 years explain on average ~23% (maximum of 44% in certain locations) of the total variance in basal melting (Fig. 3k), while ~7% (maximum of 21%) of the total variance is explained by modes that occur over 5–10 years (Fig. 3l). Multidecadal modes in basal melting are present but explain on average ~6% (maximum of 24%) of the total variance (Fig. 3m). Histograms of in-band SSA variance for the chosen time bands are shown in Supplementary Fig. 2.

### Bottom temperature interannual variability

**Fig. 4:**
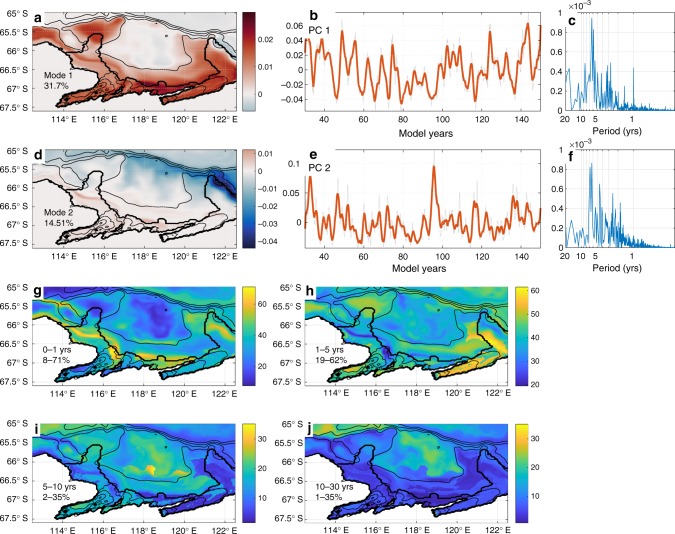
Intrinsic variability in bottom temperature. **a** Bottom temperature anomaly EOF mode 1 and **b** PC 1. **c** Spectral variance (power spectrum times frequency) for bottom temperature PC 1. **d** EOF mode 2 and **e** PC 2. **f** Spectral variance for bottom temperature PC 2. SSA in-band variance is shown for time bands **g** 0–1 years, **h** 1–5 years, **i** 5–10 years and **j** 10–30 years, with min–max explained variance shown. Thin contours show bathymetry of 500, 1500, 2000, 2500 m. Orange line in **b** and **e** is smoothed with a 1.5-yr filter, while grey line is raw data. Colour bars in **g**–**j** are scaled to the min/max variance range, as given as percentages in the lower left hand corner of each panel

Low-frequency variation in melt rate is being driven by intrinsic ocean variability. EOF analysis of bottom temperature anomaly shows that 32% of variability is explained in the shallower, coastal region (Fig. 4a, b), and has a multimodal spectrum with power in the interannual time band (Fig. 4c). Shelf break exchange (Fig. 4d, e) explains 15% of variability and temperature anomaly EOF1 has multimodal power in the interannual band (Fig. 4f). The intra-annual in-band variance in bottom temperature anomaly peaks along the ice front, explaining up to 71% of total variability (Fig. 4g), while 1–5 year modes (Fig. 4h) produce 40–60% of total variability over the continental shelf. The 5–10 year mode (Fig. 4i) explains 20–30% of total variability on the continental shelf, while the multidecadal mode (Fig. 4j) explains 20% of total variance deeper than ~500 m. Bottom temperature variability is generally aligned along bathymetric features, indicating that ocean–bathymetry interactions are a significant source of variability.

## Discussion

We show melting responds rapidly and linearly to the thickness of the relatively warm MCDW layer (Fig. 2b) present at the ice front. Cross-shelf exchange displays intrinsic variability but low correlation with melting, suggesting that intrinsic variability in melting results from local processes like wind-driven changes to the MCDW layer thermocline depth, rather than cross-shelf exchange. Comparison of the COREv1 (intrinsic ocean variability) run to a sensitivity study where the interannual intrinsic ocean variability is removed from the lateral boundary conditions, shows that indeed the variability that impacts the supply of ocean heat to the ice shelf cavity is generated within our model (see Supplementary Fig. 3). We can speculate that the non-linear ocean processes that generate such intrinsic ocean variability could include long-period baroclinic instabilities, which when subject to essentially stochastic winds, give rise to variability in ocean properties across a wide band of frequencies. This includes low-frequency modulation of the thickness of the MCDW layer on the continental shelf.

The chosen surface and lateral boundary conditions mean that any model variability with a period of greater than one year must be intrinsic ocean variability that is generated within the model, or imported through the Northern, Eastern and Western lateral boundaries (see Supplementary Figs. 4–6). Intrinsic variability in melting occurs underneath the entire ice shelf with a maximum range of ~1 m yr^−1^ (Fig. 3b). The period of variation is multimodal but has dominant periods of ~3 and ~7 years (Fig. 3f, i). The presence of intrinsic variability is weakest at the shallow ice front, which is most susceptible to seasonal sea ice and wind processes, and is stronger at depth (Fig. 3k–m). Up to 44% of 1–5 year variability and up to 21% of 5–10 year variability in melt rate results from intrinsic processes.

The in-phase response and high degree of variability that is explained by melt EOF1 suggests that cavity waters rapidly adjust to ocean heat entering this relatively small volume (Fig. 3d). Furthermore, the coherent response suggests that a reasonably representative assessment of basal melting can be obtained with few observations. The in-phase response also makes basal melting beneath TIS a good candidate for data assimilation and for parameterisation into an ice sheet model.

The high degree of variability in basal melting that results from intrinsic modes present in the 1–5 year time band (up to 44%) could contribute to the ~3–4 year period variations observed in TIS thickness^2^. Futhermore, the 5–10 year modes (explaining up to 21% of variation) likely contribute to the measured decadal period variations in flux^3^ and surface velocity^4^.

In general, we find that variability in bottom temperature occurs mainly on the shallower regions of the continental shelf (Fig. 4a), likely resulting from changes in thermocline depth in response to surface wind forcing with a peak 5–10 year periodicity (Fig. 4c). The second EOF (Fig. 4d) captures cross-shelf exchange and associated warm water pathways. Both MCDW layer thickness and cross-shelf exchange are shown to contain large low-frequency variations (Fig. 2b, c). The similar periodicity to EOF mode 1 results from a common mechanism as upwelling responds to wind forcing. The relationship between basal melting and wind stress is elsewhere demonstrated to be caused by Ekman pumping and upwelling of oceanic heat, which in turn correlates well with observed TIS surface velocity anomalies^5^. As melting underneath the Totten Ice Shelf has been identified to be driven by the relatively dense, warm and salty MCDW in models^25^ and observations^28,29^, it follows that intrinsic variability in ocean water properties near the seabed produces a response in basal melting.

Intrinsic variability has been previously identified in Antarctic sea ice patterns^10,31,32^, in the Southern Ocean^9,10^, as well as hypothesised in the ice sheet response to accumulation variability^33^ and in palaeoclimate dynamics^34^. Our results demonstrate that basal melting can vary on interannual timescales purely as a result of internally generated ocean variability, with broad implications for the identification of variability in an ice shelf/ocean system.

Interannual variability is present in ice shelf elevation change^2^, surface velocity^3–5^ and grounding line location^6^. However, it is difficult to extract the impact of intrinsic interannual variability in melt rate from these broad-scale products due to the disparate temporal and spatial scales, and relatively short observation periods. Nevertheless, the presence of interannual variability in multiple sources, with similar modes, suggests that some degree of the observed variability results from intrinsic processes.

This study has implications for observing and understanding ice shelf-ocean interaction. Basal melting can vary periodically with low-frequency variability, even in the absence of interannual atmospheric forcing. This implies that at least part of the variability associated with ice shelf thickness and consequently mass balance could arise purely through intrinsic ocean processes. This intrinsic variability is subsequently modified by external climate drivers to produce the observed record. However, to fully understand the temporal and spatial impact on ice shelf flow and thickness dynamics, a coupled ocean-ice sheet model is required.

We have highlighted the emergence of low-frequency intrinsic variability in cross-shelf flow and MCDW layer thickness, which arises through non-linear ocean processes and in response to daily-to-seasonal changes in wind stress. Basal melting beneath TIS responds linearly to MCDW thickness, but has a low correlation with cross-shelf flow, suggesting locally generated intrinsic variability influences the heat available to drive melting. This complex variability in basal melting limits the ability of short-term observational missions to draw conclusions about the long-term response to anthropogenic factors. In this sense, short oceanographic voyages and mooring deployments should be employed together with modelling studies to explore longer term trends. In regions which display significant interannual intrinsic ocean variability, long-term observational time series are critically important. Furthermore, many other ice shelves around Antarctica display some form of interannual variability (e.g. Filchner-Ronne, Amery, Shackleton and Ross^2^). The contribution of intrinsic ocean processes to the observed interannual variability ice shelves around Antarctica is yet unknown. Understanding and distinguishing this component of the signal through modelling studies is important for predicting and detecting climate change, planning observational missions that target regions of low intrinsic variability, and interpreting satellite and in situ observations.

## Methods

### Model details

To investigate the response of basal melting to intrinsic ocean variability, we ran numerical ocean model simulations of the TIS over a period of 150 years. The ocean model is based on the Regional Ocean Modelling System (ROMS)^35^ framework, with modifications to allow thermodynamic interaction between the ocean and a steady-state ice shelf^36,37^. The model domain extends 25.5° in longitude (104.5° E–130° E) and 8° in latitude (60° S–68° S), with a longitudinal resolution 1/15° and latitudinal resolution of 1/30° (Fig. 1a). Ice/ocean thermodynamics are parameterised using the three-equation formulation^38^. More details of the model setup are given in Gwyther et al.^25^ The model bathymetry is based on RTopo^39^, which provides the deep ocean and continental shelf bathymetry. RTopo does not included realistic bathymetry for TIS so we blended it with a TIS cavity bathymetry inverted from airborne gravity measurements that also includes a hypothesised trough connecting the continental shelf to an eastward extension of the TIS cavity^40^. This modelling study includes direct ocean access to the eastern extension of the TIS cavity, which satellite altimetry missions indicate hosts a surface lowering signal distinct from the deeper TIS grounding zone^41^. Ice draft was determined with airborne radar sounding measurements acquired by the ICECAP (International Collaborative Exploration of the Cryosphere through Aerogeophysical Profiling) consortium.

The first simulation has surface wind forcing from COREv1 which corresponds to repeating normal-year (1995) momentum fluxes^21^. Surface heat and salt fluxes are taken from the 1995 record of sea ice formation from Special Sensor Microwave Imager (SSM/I) satellite observations^42^. This year is chosen as it represents a normal climate year, and unlike CORE forcing, SSM/I observations better capture the timing, magnitude and spatial extent of sea ice and polynya formation^42^. Lateral forcing consisted of downscaled results from a general circulation model^10^, which itself was forced with repeated normal-year COREv1^21^ forcing. The lateral boundary conditions are applied interannually, so as to capture intrinsic oceanic variability in Southern Ocean properties (Supplementary Figs. 4 and 5), in particular, the 20-year South Pacific Intrinsic Mode^10^, but no other modes of variability (e.g. coupled land–ice–ocean-atmosphere processes such as climate modes). If a repeated normal-year forcing were applied to the lateral boundary conditions, then slow oceanic modes would not be excited and in effect, the ocean variability spectrum would be truncated. Here we are concerned with variability in melt rate rather than the mean value, and so the choice of lateral boundary conditions is valid, despite the reduced mean melt rates. The mean value is low due to cooler conditions in the Southern Ocean, a common model bias in ocean GCMs forced with COREv1 conditions.

The second run (COREv2) of 120 years was forced with two periods of 1949–2007 interannual climate forcing. Surface wind forcing is from the COREv2 1949–2007 air–sea flux data set^22^, while surface heat and salt fluxes are composed of the climatology of surface heat and salt fluxes from SSM/I observations for 1995^42^ but scaled by the E − P (evaporation minus precipitation) fields for 1949–2007 from COREv2. These data sets are combined in this way to provide long-term estimates of polynya activity and sea ice formation, which are not obtainable from a data set of comparable length. The lateral boundary forcing is analogous to that in the first simulation, but now the global ocean circulation model^10^ is itself forced with the COREv2 1949–2007 interannually varying air–sea fluxes^22^. As a result, the lateral boundary forcing is representative of the oceanic response to the large-scale atmospheric climate variability including the complete forcing spectrum with all natural, intrinsic, internal (e.g. climate modes) and anthropogenic forcing components. The first period of 60 years is considered spin-up.

A sensitivity study investigating whether intrinsic variability in melt rate originates locally or is imported through the boundary conditions was conducted (see Supplementary Fig. 3). In this run, we force with the same normal-year atmospheric conditions as previously. However, we have changed the lateral boundary conditions to remove the interannual intrinsic variability, replacing them with a repeated seasonal cycle calculated as the climatology of the previously-used interannual COREv1 intrinsic variability forcing^10^. These new boundary conditions, where we removed any interannual variability, enable an estimate of the variability intrinsic to the ROMS domain.

### Analysis techniques

For all analyses, the first 30 years of time series of melt and bottom temperature, which contain the model spin-up signal, are excluded. The resulting time series are detrended with a linear fit.

Signal-to-noise ratio is calculated as the ratio of variances *σ*^2^, calculated as

1 $${\mathrm{SNR}} = \frac{{\sigma _{{\mathrm{COREv}}2}^2}}{{\sigma _{{\mathrm{COREv}}1}^2}},$$

where $\sigma _{{\mathrm{COREv}}2}^2$ is the square of the standard deviation of the melt rate anomaly for the interannually varying run with COREv2 forcing, while $\sigma _{{\mathrm{COREv}}1}^2$ is the square of the standard deviation of the melt rate anomaly for the intrinsic variability-only run with COREv1 forcing.

Cross correlations of MCDW depth and volume flux with melt rate were calculated and a lag of 0 months is found to be optimal. As analysis is computed on monthly averages, a short lag or lead of less than 1 month is possible.

Empirical orthogonal function (EOF) analysis is used to decompose a time series into orthogonal spatial patterns (EOFs) along with the associated time series or principal components (PCs) and the variance explained by each mode^43^. While EOF analysis can provide useful decomposition of data, it has limitations such as domain dependence. While the leading mode(s) may often be physical, successively higher order modes, which explain less of the remaining variance, are unphysical as a consequence of the mode orthonormalisation.

Consequently, we employ singular spectral analysis (SSA) to express the original time series as a linear combination of components derived from singular value decomposition of the (time) lagged covariance matrix with maximum lag *M*. The resulting modes together sum to the original time series, while the slowest varying mode represents the non-linear trend with (time) variability of the order of the chosen embedding dimension *M* or longer. The decomposition is data driven (based on smaller, lagged portions of the original time series) and does not require projection on predefined basis functions, as required for example with Fourier transform methods. SSA readily provides non-linear smoothing of the original time series with the degree of smoothing controlled by *M*^12,30^. The SSA in-band variance shows the percentage of total variance present in the chosen time band bounded by predefined embedding dimension values, i.e. 0–1, 1–5, 5–10 and 10–30 years as in Figs. 3j–m and 4g–j.

Plots made with assistance from the Antarctic Mapping Tools for MATLAB toolbox^44^ and cmocean^45^ colourmaps.

### Data availability

Model output that supports the findings of this study are available from the corresponding author on request.

## Electronic supplementary material

Supplementary Information
