# Supplementary material for: Intrinsic processes drive variability in basal melting of the Totten Glacier Ice Shelf
Source: Nat Commun. 2018 Aug 7;9:3141. doi: 10.1038/s41467-018-05618-2 (PMC6081466; doi:10.1038/s41467-018-05618-2)
Supplement: Supplementary file 1 — Supplementary Information [file 41467_2018_5618_MOESM1_ESM.pdf]

Supplementary Information

Intrinsic processes drive variability in basal melting of the Totten Glacier Ice Shelf

Gwyther et al.

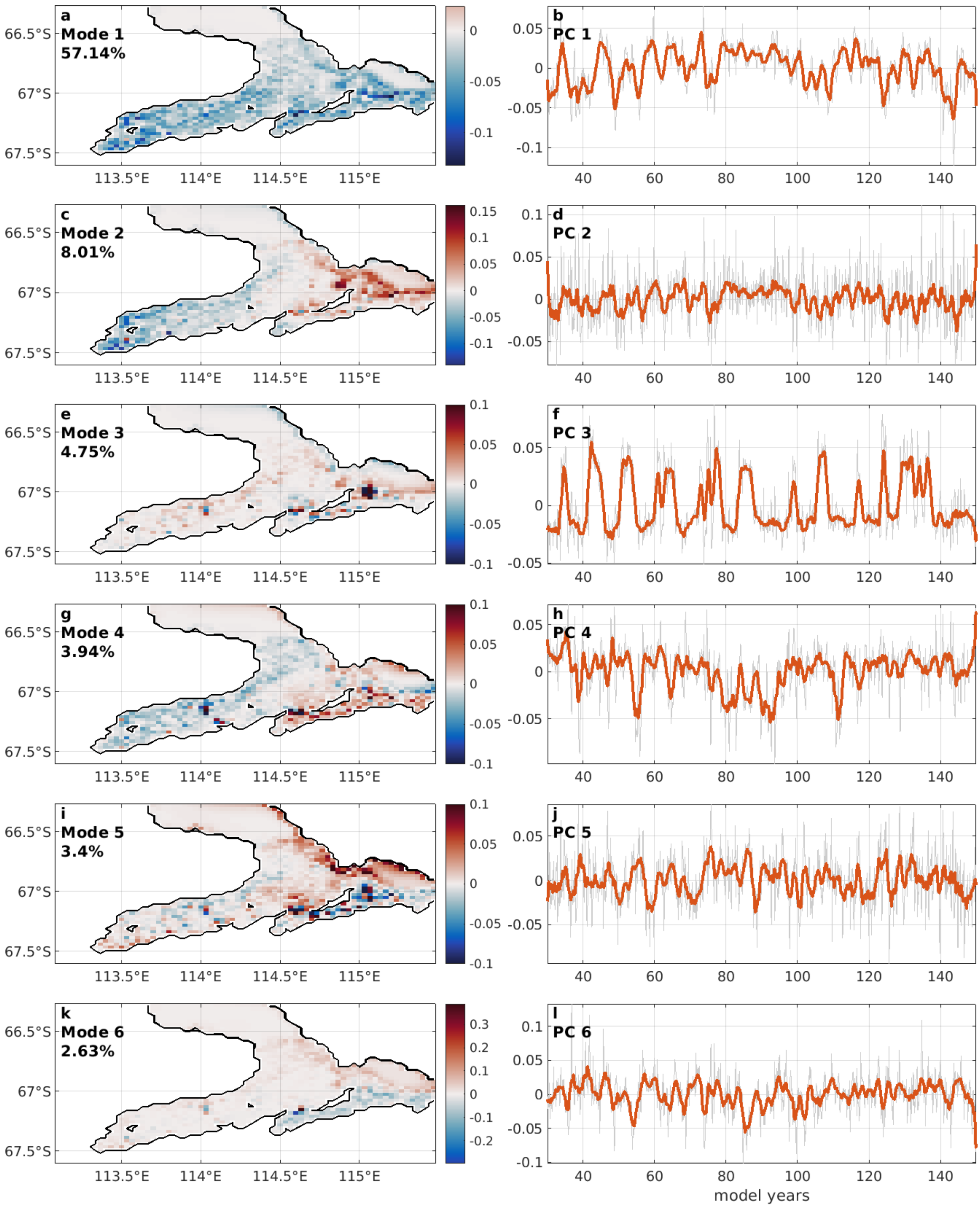

Supplementary Figure 1 | First 6 EOF modes of basal melting. a,c,e,g,i,k First 6 EOF modes are shown for the Totten Ice Shelf basal melting. **b,d,f,h,j,l** First 6 PCs are shown for the time series of basal melting. Together, the first 6 modes explain nearly 80% of total melt variability.

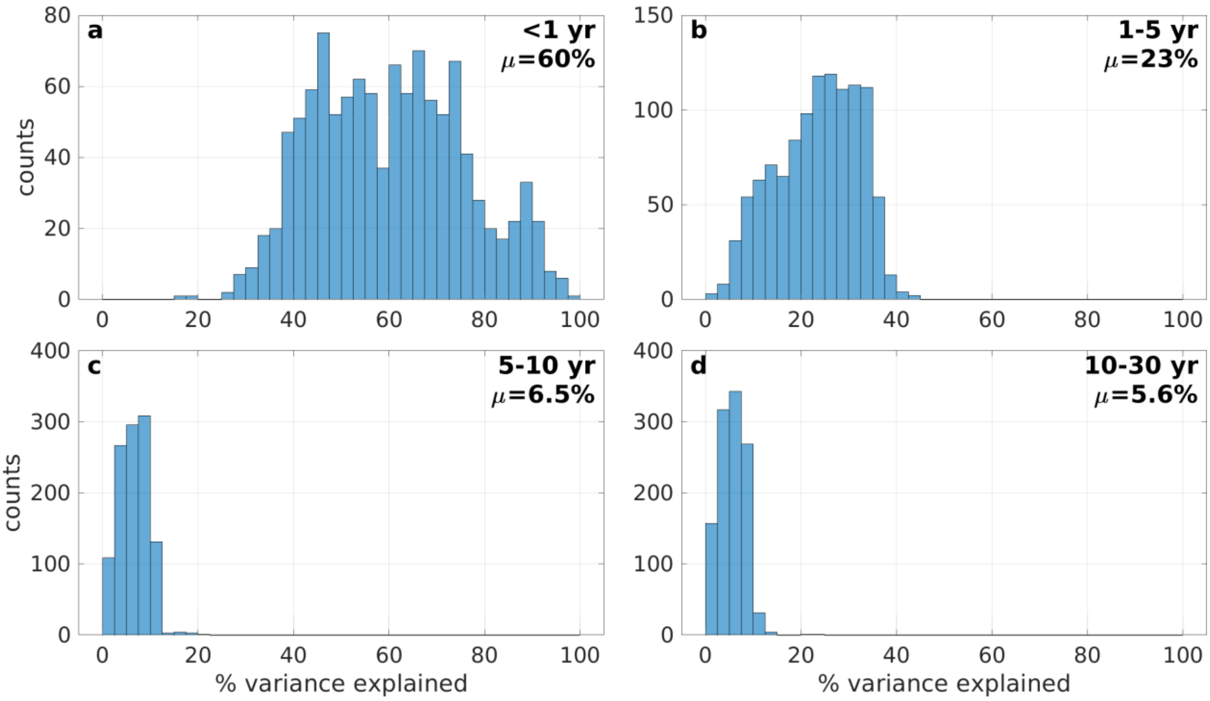

Supplementary Figure 2 | Histograms of in-band variance for melt rate. Histograms show the distribution of variances explained by the in-band SSA for **a** <1 yr, **b** 1-5 yrs, **c** 5-10 yrs, and **d** 10-30 yrs, for the melt rate anomaly shown in Figure 3. The mean of each distribution is shown below the time band.

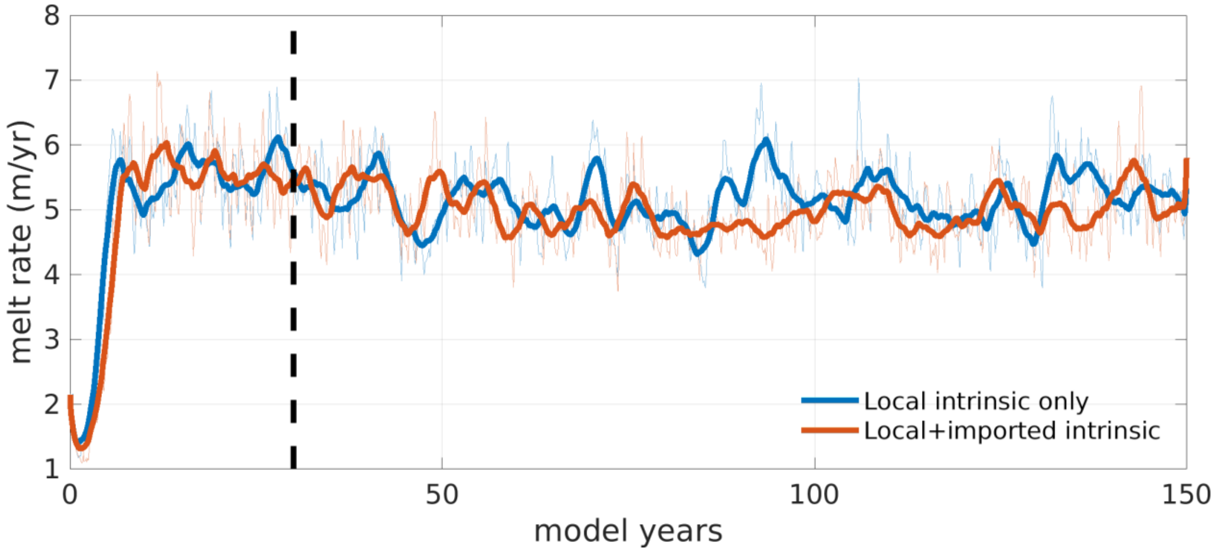

Supplementary Figure 3 | Melt rates responding to local and imported intrinsic variability. Area average melt rate for COREv1 repeated-normal year forcing with intrinsic ocean variability imported through the boundary conditions (local+imported intrinsic; orange line), and a sensitivity study with identical COREv1 forcing but without the interannual lateral boundary forcing (Local intrinsic only; blue line). The lateral boundary conditions are changed to remove the interannual intrinsic variability, replacing them with a repeated seasonal cycle calculated as the climatology of the previously-used interannual COREv1 intrinsic variability forcing (O'Kane et al., 2013). Raw data (light coloured lines) is smoothed with a 3-yr filter. Dashed black line demarcates the 30-year spinup period not used for analysis.

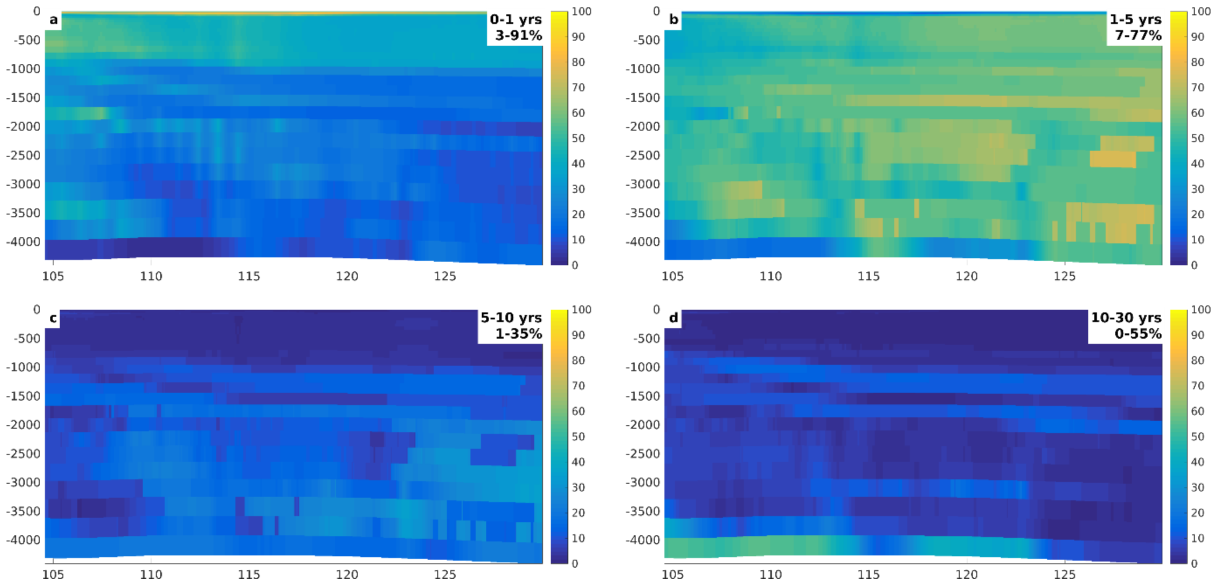

Supplementary Figure 4 | In-band variance of Northern boundary temperature anomaly. Variance explained in temperature anomaly at the Northern boundary is shown for SSA time bands **a** 0-1 years, **b** 1-5 years, **c** 5-10 years and **d** 10-30 years, with min-max explained variance shown. Intrinsic variability is displayed in all interannual bands.

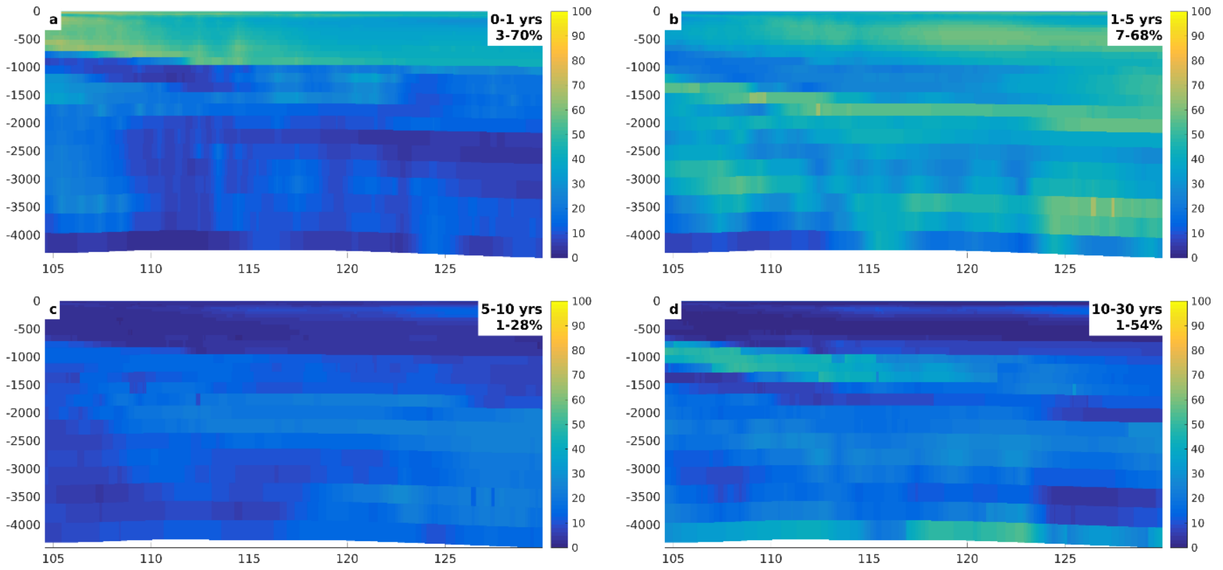

Supplementary Figure 5 | In-band variance of Northern boundary salinity anomaly. Variance explained in salinity anomaly at the Northern boundary is shown for SSA time bands **a** 0-1 years, **b** 1-5 years, **c** 5-10 years and **d** 10-30 years, with min-max explained variance shown. Like temperature anomaly (Supplementary Figure 4), intrinsic variability is displayed in all interannual bands.

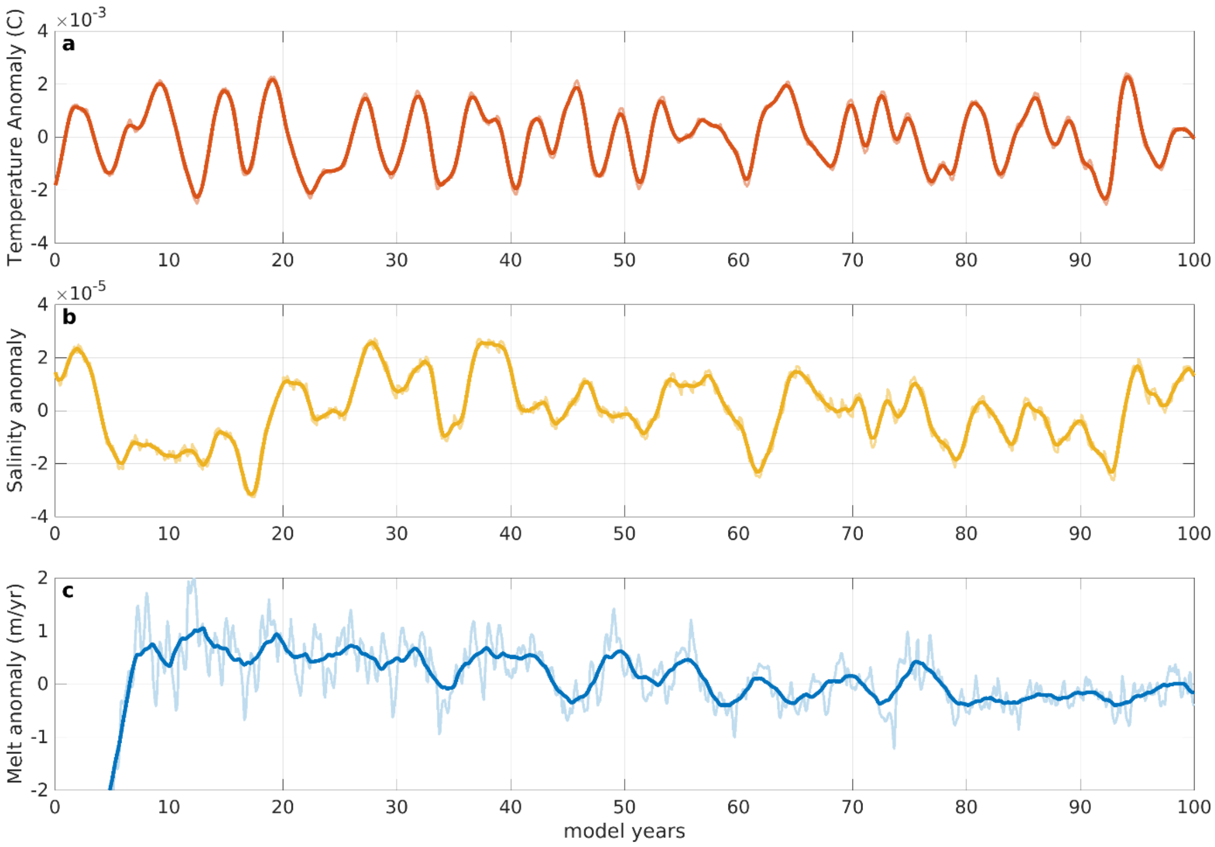

Supplementary Figure 6 | Time series of Northern boundary properties. The average time series of Northern boundary **a** temperature anomaly and **b** salinity anomaly below 1000 m. Both properties display interannual, intrinsic variability, but do not correlate with **c** melt rate anomaly. The average is calculated below 1000 m to remove the influence of the seasonal cycle shown in Supplementary Figure 4 and 5.
